# Statin Induced Myopathy and Myalgia: Time Trend Analysis and Comparison of Risk Associated with Statin Class from 1991–2006

**DOI:** 10.1371/journal.pone.0002522

**Published:** 2008-06-25

**Authors:** Mariam Molokhia, Paul McKeigue, Vasa Curcin, Azeem Majeed

**Affiliations:** 1 Department of Epidemiology & Population Health, London School of Hygiene & Tropical Medicine, London, United Kingdom; 2 Public Health Sciences, University of Edinburgh Medical School, Edinburgh, United Kingdom; 3 Department of Computing, Imperial College London, London, United Kingdom; 4 Department of Primary Care and Social Medicine, Imperial College, London, United Kingdom; University of Groningen, The Netherlands

## Abstract

**Background:**

Statins are widely used as a cholesterol lowering medication, reduce cardiovascular mortality and morbidity in high risk patients; and only rarely cause serious adverse drug reactions (ADRs). UK primary care databases of morbidity and prescription data, which now cover several million people, have potential for more powerful analytical approaches to study ADRs including adjusting for confounders and examining temporal effects.

**Methods:**

Case-crossover design in detecting statin associated myopathy ADR in 93, 831 patients, using two independent primary care databases (1991–2006). We analysed risk by drug class, by disease code and cumulative year, exploring different cut-off exposure times and confounding by temporality.

**Results:**

Using a 12 and 26 week exposure period, large risk ratios (RR) are associated with all classes of statins and fibrates for myopathy: RR 10.6 (9.8–11.4) and 19.9 (17.6–22.6) respectively. At 26 weeks, the largest risks are with fluvastatin RR 33.3 (95% CI 16.8–66.0) and ciprofibrate (with previous statin use) RR 40.5 (95% CI 13.4–122.0). AT 12 weeks the differences between cerivastatin and atorvastatin RR for myopathy were found to be significant, RR 2.05 (95% CI 1.2–3.5), and for rosuvastatin and fluvastatin RR 3.0 (95% CI 1.6–5.7). After 12 months of statin initiation, the relative risk for myopathy for all statins and fibrates increased to 25.7 (95% CI 21.8–30.3). Furthermore, this signal was detected within 2 years of first events being recorded. Our data suggests an annual incidence of statin induced myopathy or myalgia of around 11.4 for 16, 591 patients or 689 per million per year.

**Conclusion:**

There may be differential risks associated with some classes of statin and fibrate. Myopathy related to statin or fibrate use may persist after a long exposure time (12 months or more). These methods could be applied for early detection of harmful drug side effects, using similar primary care diagnostic and prescribing data.

## Introduction

Existing approaches to detection of rare but serious adverse drug reactions (ADRs) have limitations. Such associations are often too rare to be detected in early clinical trials, and may not appear until after a drug has been more widely released. Post-marketing surveillance relies on the vigilance of doctors (spontaneous reporting), or on prescription event monitoring of relatively small numbers of exposed individuals over a limited period, without appropriate controls. Failure to detect adverse drug reactions early increases the risks associated with drug development, with consequent higher drug costs, lower rates of innovation, and greater healthcare costs. Consequently, current systems of detection, verification and quantification of ADRs are disparate, reliant mainly on reports reaching the medical literature.[1]

Figures from the National Audit Office show that the primary care drugs bill in England increased from £4.0 billion in 1996 to £8.2 billion in 2006, with approximately 10 million statin prescriptions in 2006.[2] Statins are widely and safely used as a cholesterol lowering medication and have been shown to significantly reduce cardiovascular mortality and morbidity in patients with high risk of cardiovascular disease (CVD);[3], and cause serious ADRs only in a very small minority of patients. Adverse drug reactions (ADRs) are estimated to affect around 7% of patients or hospital admissions at an annual cost of around £380 million in England.[4] A recent article has reviewed the safety of statins in clinical practice, including myalgia and myopathy, from numerous clinical trials. However, risks may be underestimated as these are rare events which may not become apparent in smaller trials and it is difficult to assess risk associated with specific drugs.[5] Further studies have identified significant risks of myopathy and myositis associated with statins and fibrates using different study designs, in both a US managed care group[6] and a UK general practice population.[7]

### Use of primary care databases for ADR detection

The computerization of primary care in the UK may help in the development of new and more rapid methods of detecting adverse drug reactions in clinical practice. The computerisation of primary care has led to the creation of primary care databases with longitudinal medical records and drug prescription data covering several million people.[8] In principle, it is possible to exploit these databases for a more direct approach to the detection of associations between drugs and adverse events, as ascertainment of adverse events can be almost complete in practices with good systems for collecting diagnostic data.

Because primary care databases follow individuals before, during and after exposure to drugs, an alternative approach to control for confounding can be used, based on comparing the rate of adverse events while exposed to a drug with the rate of adverse events in the same individuals while they are unexposed to the drug (case-crossover design). This approach was first developed to study the effects of transient, short-term exposures on the risk of acute events[9] and has been used in studies of adverse effects associated with vaccines[10] and risk of MI after acute respiratory infection,[11] and more recently increasingly in pharmacovigilance studies.[12]–[19] Because each individual is included both as a case and a control, this design considerably reduces confounding by co-morbidity.

Examples of primary care databases in the UK include the General Practice Research Database, The Health Information Network (THIN), QRESEARCH and MediPlus. The size of these databases gives them sufficient power to detect even rare adverse drug reactions. Key objectives for this study were to develop a methodology for exploiting primary care databases for signal detection and identify how soon an ADR can be identified using prescribing and medical data.

## Methods

### Study design

Case-crossover retrospective study from 1991–2006

### Data sources

We used The Health Improvement Network (THIN) and MediPlus databases for this study. These databases classify drugs by the ATC (Anatomical Therapeutic Chemical classification) system or equivalent such as the British National Formulary coding (BNF), and code morbidity using READ codes. The databases cover an active population of about 5 million people, and many patients have a follow-up period of over ten years.

The information collected in THIN and MediPlus included for each patient in the database: comprises (i) consultations coded with READ codes; (ii) measurements including laboratory test results, blood pressure, height and weight; (iii) details of drug prescriptions and (iv) demographic items. The databases also include diagnoses coded following hospital discharge and outpatient encounters. Quality measures were also available which allow selection of complete records with respect to denominator, prescribing and demographic data. The accuracy of drug prescription data was high, as prescriptions were generated by general practitioners using the VISION software from which the THIN and GPRD databases are generated. Ethical approval for this study was obtained from the LSHTM ethics committee, the MediPlus Independent Scientific and Ethics Advisory Committee, and the NHS South-East Multi-centre Research Ethics Committee (MREC) for the THIN scheme.

### Statistical methods and power calculations

The statistical analyses investigate how causal relationships can be inferred from the temporal relationship between drug exposure and outcome, and the size of the effect. A key advantage of the case-crossover design is that confounding by co-morbidity is reduced, as each individual acts as their own control.

Sample size calculations show that adequate power to detect associations can be achieved with relatively small numbers of events occurring while individuals are exposed, as the rate ratios for ADR risk are typically large. If the number of events while unexposed is large compared with the number *a* of events while exposed, the standard error of the log rate ratio is simply *√(1/a).* Even if the proportion of time exposed (among all individuals who experience adverse events) is as high as 5%, this approximation holds well. The minimum detectable log rate ratio for a Type 1 error rate of 2 α and a Type 2 error rate of β is then (z_α_+z_β_)√(1/a), where z_α_ and z_β_ are the quantiles of the standard normal distribution. To allow for multiple testing, we have set α = 0.0001 and β = 0.1. Then with 20 adverse events while exposed (plus a much larger number of adverse events while not exposed) we can detect a rate ratio of 3.6, and with 50 adverse events while exposed we can detect a rate ratio of 2.4.

With more than 50 million person-years, there are enough events to examine any class of drug-induced ADR that has a population incidence of at least 1 per million per year.

For all patients in the study the inclusion was the first ever myopathy code after registration (see Appendix S1). We excluded any patients who had never had a statin prescription. Patients were also excluded if they received steroids within 2 weeks of the myopathy event, were receiving anti-retroviral therapy and had been diagnosed with any rheumatic disease. We then examined start of new statin, change of statin prescriptions, or increase in statin dose in the 12 weeks (and for other exposure periods) prior to the myopathy event code. Therefore myopathy events were classified as “exposed” if they occurred within 12 weeks of new/change of statin and “non-exposed” if not on statin at time of myopathy event (or had been taking statin for greater than cut off for exposure time). Numbers of events and time periods of study were calculated during i) exposure and ii) non exposure. We calculated denominator periods for exposed and non exposed groups by year, myopathy code and drug class. RR were calculated as the ratio of numbers of events when exposed to the number of events when unexposed, taking into account the relevant denominator data of “exposed” and “unexposed” times. SE and 95% CI were calculated using standard methods, as described earlier. A window of 6 months (to exclude repeat consultations with the same READ code relating to the initial diagnosis) was used to examine possible re-challenges which were defined as new recorded code for myopathy associated with statin use in the preceding 12 weeks. Altman and Bland tests for interaction were applied to determine if the difference in RR was significant between statin classes and periods of study.[20]

### Drug exposure data

A list of the corresponding British National Formulary (BNF) drug codes was assembled for classes of statin, and each class of adverse events. Data relevant to testing for association between the statin class and the adverse event class included episodes of exposure to statins, tables of exposed and unexposed individuals with morbidity and demographic data, and tables of all adverse events in this class. For each statin class, and for each class of adverse events, data was carefully checked for inconsistencies; for instance, errors in the prescription record were identified as outliers in the distribution of calculated daily dosages. Varying definitions of exposure (12, 26 and 52 weeks) were defined, for example, as new statin started within last 12 weeks, change of statin or dose increased within the last 12 weeks.

### Morbidity data

Morbidity data were available in the form of READ codes, with additional health data including clinical and laboratory measurements also available. READ codes include diagnoses, symptoms, demographic variables, and other types of information. However, although the drug prescribing data in these databases was highly consistent, the diagnostic coding was less consistent and required programming of additional validation checks. Specifically, the READ classification does not necessarily correspond to disease entities, and additional work was necessary to define criteria for classes of adverse events such as myopathy, myalgia and myositis which were included in the analyses. Additionally we selected READ codes for creatinine kinase measurements and included cases who had elevations at >10 times the upper limit of normal 100 IU/ml in females and 150 IU/ml in males, although not all patients with myopathy have elevated CK values.[21] Codes included identifying co-morbidities and potential confounders (for example cases with codes for rheumatic diseases and steroid use were excluded).

### Data management

The workflow data processing methodology used in the project was based on the InforSense KDE workflow system.[22] The software originated from the Grid Computing and Service Computing developed within the Discovery Net, UK e-Science Pilot Project[23].

**Figure 1:**
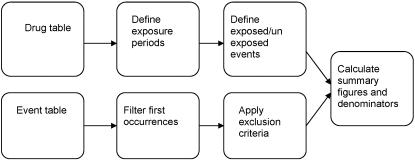
Example of InforSense workflow methodology to derive summary statistics.

The InforSense KDE infrastructure has been designed and implemented on the basis of a scientific workflow, for composition of data analysis tools and resources. The approach supports grid-based data analytics that require integration of diverse and distributed sources and enables remote access to the resulting analysis. It also supports more traditional workflow functionality that can be used to route tasks between different users. An overview of the InforSense architecture and workflow methodology, used in preparing the data files for analyses are shown in Figure 1.

The methodology developed for this study consists of a data integration strategy for the THIN relational schema, and reusable modules for patient selection, event selection, event correlation and exposure definition. All these have been initially defined upon flat text files, with a view to allow future direct migration to a high-performance relational database (e.g. Oracle 10 g). Therefore, the data processing operations have been designed independently from the underlying storage.

Advantages of this methodology include flexibility, which allows parameterisation of the key reusable features of the workflow, such as size of the exposure window, dealing with repeated prescriptions, and handling incomplete dosage information. The workflow also provides a basis for further studies on other clinical and prescription datasets.

## Results

The data extracted were based on 77,240 statin users from IMS MediPlus between 1991–2004 and 16,591 statin users from THIN data between 1991–2006. THIN data contained 516, 331 episodes of independent types of drug use (including different dosages of formulations). Table 1 shows descriptive data of patients with myopathy overall and those with myopathy taking statin therapy. Patients with myopathy receiving statins were older with a slight male excess (53.2%). Table 2 gives a breakdown of statin prescriptions by year from 1991–2005 using the THIN database. Table 3: shows statin and fibrate prescriptions by class of drug and year from 1990–2005 using THIN database.

**Table 1:** Descriptive data on all patients from THIN with myopathy or myalgia READ code.

	All patients with myopathy
	N = 27689
	n (%)
Mean [SD] age at event in years	47.8 [18.9]
Aged over 65 years	5565 (20.1%)
Female	15752 (56.9%)
Smokers	8828 (31.9%)
Mean CK [SD] where recorded n = 4318	125 [236]; range 0–7550
Mean BMI baseline [SD] kg/m^2^ n = 23659	26.2 [6.0]

	Patients with myopathy and recent statin use n = 4258
Mean [SD] age at event in years	64.7 [10.8]
Aged over 65 years	2146 (50.4%)
Female	1993 (46.8%)
Smokers	1331 (31.3%)
Mean CK [SD] where recorded n = 2135	127 [142]; range 0–3597
Mean BMI baseline [SD] kg/m^2^ n = 4057	28.2 [5.8]

**Table 2:** Statin prescriptions by year using THIN database 1990–2005.

Year	Statin & Fibrate prescriptions	Statin prescriptions	Fibrate prescriptions
1990	1009	203	806
1991	2190	865	1325
1992	2915	1368	1547
1993	3910	1951	1959
1994	4881	2556	2325
1995	6826	3976	2850
1996	10611	7201	3410
1997	15053	11650	3403
1998	20875	17806	3069
1999	28720	25746	2974
2000	39039	35980	3059
2001	50923	47746	3177
2002	65254	61861	3393
2003	81461	77806	3655
2004	97925	94223	3702
2005	87352	84109	3243

**Table 3:** Statin & fibrate prescriptions by drug class and year using THIN database 1990–2005*.

Year	atorvastatin	cerivastatin	fluvastatin	pravastatin	rosuvastatin	simvastatin	bezafibrate	fenofibrate	ciprofibrate
1990				2		201	742	8	
1991				183		682	1249	31	
1992				318		1050	1451	71	
1993				482		1469	1745	103	89
1994			51	637		1868	1856	192	264
1995			290	670		3016	2111	275	445
1996			453	1183		5565	2463	337	593
1997	783	157	964	1792		7954	2438	356	596
1998	3187	1024	974	2423		10198	2156	346	553
1999	6236	2273	1179	3439		12619	2037	416	512
2000	10750	3583	1296	4813		15538	2041	520	493
2001	16386	3162	2210	6287		19701	2025	674	478
2002	23743		2936	8696		26486	2112	856	425
2003	31092		2668	10061	1171	32814	2223	975	457
2004	37324		1985	9529	4361	41024	2056	1233	413
2005	33460		1268	6658	4227	38496	1670	1237	336

* data for clofibrate omitted.

Tables 4 & 5 using independent data from IMS and THIN, show that using a case-crossover design has the potential to pick up ADRs such as myopathy or myalgia with statin use, with increased rates in exposed individuals. We have included first occurrences of morbidity only, so that individuals with chronic myalgia or myositis have less influence on the results. The large numbers with “myalgia unspecified” may include some with myopathy, as this shows a strong association with statin exposure. The IMS data showed large rate ratios (RR's) for all myopathy codes and statin exposure; for all codes apart from polymyositis the rate ratio was 8.2 (95% CI 4.1–16.3). The THIN data show a similar magnitude of RRs associated with statin exposure, the combined result for all myopathy codes and statin exposure (within last 12 weeks) was 10.6 (95% CI 9.8–11.4). The magnitude of the RR with known elevated CK levels was 8.0 (95% CI 5.8–11.1), although CK values were available on only 5% unexposed and exposed cases. Using the READ code analyses (THIN), largest risks were associated with the term “muscular rheumatism” and “fibrositis unspecified” RR 22.3 (95% CI 6.8–72.6) and 29.1 (95% CI 15.2–56.1) respectively (part of composite RR given in Table 5).

**Table 4:** Case-crossover comparison of myopathy/myalgia based on 77240 statin users extracted from the IMS MediPlus database (1991–2004) using 12 week cut-off for exposure.

Code Text	Exposed Events	Un-exposed Events	Rate Ratio (95% CI)
	598 p-yrs	11,206 p-yrs	
Myopathy-all codes Toxic myopathy; proximal myopathy; myopathy unspecified; Myopathy or muscular dystrophy unspecified	9	19	8.9 (3.5–22.4)
Fibromyalgia	28	59	8.9 (4.2–18.9)
Myalgia/myositis –all specified codes	10	19	2.3 (4.1–23.7)
Myalgia/myositis unspecified	156	409	7.2 (3.4–15.1)
Myalgia unspecified	107	205	9.8 (5.1–18.8)
Myositis unspecified	4	9	8.3 (2.5–27.5)
**Total myalgia/myositis/myopathy**	**314**	**720**	**8.2 (4.1–16.4)**
Acute renal failure due to rhabdomyolysis	0	2	-
**All except polymyositis**	**314**	**722**	**8.2 (4.1–16.3)**

The code specific RR are calculated based on denominators which reflect exposed and unexposed time for relevant code text; the total RR is calculated using total exposed and non exposed times (all codes).

**Table 5:** Case-crossover comparison of myopathy/myalgia based on 16,591 users extracted from the THIN database (1991–2006) using 12 week cut off for exposure.

Code Text	Exposed Events	Un-exposed Events	Rate Ratio (95% CI)
	204 p-yrs	592 p-yrs	
Muscle ligament or fascia disorder	9	2	10.8 (2.3–49.8)
Intercostal myalgia Fibromyalgia	145	74	14.2 (10.7–18.8)
Rheumatism and/or fibrositis unspecified; muscular rheumatism; rheumatic pain	148	45	18.4 (13.2–25.8)
`Myalgia unspecified	1632	427	9.8 (8.9–11.0)
Myositis unspecified	44	9	9.6 (4.7–19.7)
Myalgia/myositis unspecified	1	2	2.4(0.2–26.2)
Muscle pain	1188	314	9.8 (8.7–11.1)
CK level >1500 IU/L M and >1000 IU F	173	45	8.0 (5.8–11.1)
**All myalgia/myositis**	**3340**	**918**	**10.6 (9.8–11.4)**

The code specific RR are calculated based on denominators which reflect exposed and unexposed time for relevant code text; the total RR is calculated using total exposed and non exposed times (all codes).

Tables 6–8 compares events (all myopathy codes) across classes of statins and fibrates, based on different exposure times, applying the same exclusion criteria. At 26 weeks exposure, the greatest risks for myopathy are with fluvastatin RR 33.3 (95% CI 16.8–66.0) and ciprofibrate RR (40.5 95% CI 13.4–122.0). The RR for cerivastatin (which was withdrawn in 2001) was 24.7 (95% CI 11.3–54.1). The differences between cerivastatin and atorvastatin RR for myopathy were found to be statistically significant at 12 weeks RR 2.05 (95% CI 1.2–3.5), but not at 26 weeks and for rosuvastatin and fluvastatin at 12 weeks and 26 weeks RR 3.0 (95% CI 1.6–5.7) and RR 3.4 (95% CI 1.2–8.8), respectively. At 12 weeks, further differences in RR were identified for atorvastatin versus fluvastatin and pravastatin RR 0.39 (95% CI 0.24–0.63) and 0.53 (95% CI 0.40–0.70); and for simvastatin versus fluvastatin and pravastatin RR 0.44 (95% CI 0.27–0.72) and 0.61 (95% CI 0.46–0.79). At 26 and 52 weeks exposure, risk of myopathy/myalgia *increases* with statin exposure RR 19.9 (95% CI 17.6–22.6) and 25.7 (95% CI 21.8–30.3) respectively, compared with 12 week exposure. Data was analysed for fibrate class alone (12 week exposure) with RR as follows: bezafibrate 15.2 (95% CI 6.3–36.7), fenofibrate 12.2 (95% CI 2.7–55.9), ciprofibrate 2.4 (95% CI 0.2–22.6). The overall risk for myalgia/myopathy with all fibrate class (without statin co-prescriptions) was 12.8 (95% CI 6.3–25.9).

**Table 6:** Case-crossover comparison of myopathy/myalgia based on 16,591 users extracted from the THIN database (1991–2006): Event rates using 12 week cut off for exposure.

Class of Drug	Exposed Events	Un-exposed Events	Rate Ratio 12 weeks	Standard error of RR
	204 p-years	592 p-yrs		
Atorvastatin	1170	314	8.3 (7.4–9.4)	0.06
Cerivastatin	45	21	17.0 (10.1–28.5)	0.26
Fluvastatin	79	22	21.5 (13.4–34.4)	0.24
Pravastatin	313	78	15.7 (12.3–20.1)	0.13
Rosuvastatin	108	29	7.1 (4.7–10.7)	0.21
Simvastatin	1519	404	9.5 (8.5–10.6)	0.06
**All statins**	**3234**	**868**	**10.0 (9.3–10.8)**	0.04
**Ever use of statin with the following fibrate**				
Bezafibrate	62	34	18.4 (12.1–27.9)	0.21
Fenofibrate all	32	9	6.2 (3.0–13.0)	0.38
Ciprofibrate	12	7	32.0 (12.6–81.3)	0.48
**All fibrates**	106	50	**18.7 (13.3–26.1)**	0.17
**ALL statins & fibrates (95% CI)**	**3340**	**918**	**10.6 (9.8–11.4)**	0.04

**Table 7 pone-0002522-t007:** Case-crossover comparison of myopathy/myalgia based on 16,591 users extracted from the THIN database (1991–2006): Event rates using 26 week cut off for exposure.

Class of Drug	Exposed Events	Un-exposed Events	Rate Ratio 26 weeks	Standard error of RR
	350 p-years	446 p-yrs		
Atorvastatin	1401	83	15.2 (12.2–19.0)	0.11
Cerivastatin	59	7	24.7 (11.3–54.1)	0.40
Fluvastatin	92	9	33.3 (16.8–66.0)	0.35
Pravastatin	361	30	25.8 (17.8–37.4)	0.19
Rosuvastatin	128	9	9.9 (5.0–19.4)	0.34
Simvastatin	1825	98	19.5 (15.9–23.9)	0.10
**All statins**	**3866**	**236**	**19.1(16.7–21.8)**	0.07
**Ever use of statin with the following fibrate**				
Bezafibrate	82	14	25.4 (14.4–44.8)	0.29
Fenofibrate all	39	2	9.0 (2.2–37.1)	0.73
Ciprofibrate	15	4	40.5 (13.4–122.0)	0.56
**All fibrates**	136	20	**27.1 (17.0–43.4)**	0.24
**ALL statins & fibrates (95% CI)**	**4002**	**256**	**19.9 (17.6–22.6)**	**0.06**

**Table 8:** Case-crossover comparison of myopathy/myalgia based on 16,591 users extracted from the THIN database (1991–2006): Event rates using 52 week cut off for exposure.

Class of Drug	Exposed Events	Un-exposed Events	Rate Ratio 52 weeks	Standard error of RR
	419 p-years	377 p-yrs		
Atorvastatin	1448	36	21.6 (15.5–30.0)	0.17
Cerivastatin	62	4	29.5 (10.7–80.9)	0.52
Fluvastatin	93	8	35.6 (17.3–73.4)	0.37
Pravastatin	371	20	29.9 (19.1–46.9)	0.23
Rosuvastatin	135	2	14.9 (3.7–60.2)	0.71
Simvastatin	1864	59	24.5 (18.9–31.8)	0.13
**All statins**	**3973**	**129**	**24.9 (20.9–29.7)**	0.09
**Ever use of statin with the following fibrate**				
Bezafibrate	85	11	26.6 (14.2–49.9)	0.32
Fenofibrate all	40	1	9.8 (1.3–71.2)	1.01
Ciprofibrate	16	3	37.7 (11.0–129.3)	0.63
**All fibrates**	141	15	**29.0 (17.0–49.4)**	0.27
**ALL statins & fibrates (95% CI)**	**4114**	**144**	**25.7 (21.8–30.3)**	**0.08**

Using a 26 week exposure cut-off, the number of exposed events 4002 in 350 years in 16, 591 patients suggests an annual incidence of around 11.4 for 16, 591 patients or 689 per million per year, using THIN data.

### Temporal trends and re-challenge

To assess if there was any confounding by temporality; data were analysed in 3 time periods. Using a 26 week exposure period the RR showed possible evidence of a secular decrease across each of the time periods: 1995–8;1999–2002 and 2003–5; 28.7 (95% CI 15.4–53.4); 21.2 (95% CI 16.8–26.9) and 18.8 (95% CI 16.1–21.9) respectively. This pattern was similar using other exposure cut offs for 12 and 52 weeks. The difference in RR from 1995–98 compared with 2003–5 (26 week data) was found to be statistically significant RR 1.53 (95% CI 1.2–2.9), and was similar for different periods of exposure, RR 1.79 (95% CI 1.2–2.8), for 12 week exposure. Re-challenge data revealed 2 statin associated myopathy events following a drug re-challenge after 6 months or more from the primary episode, with no intervening coding listed for myopathy events.

**Figure 2:**
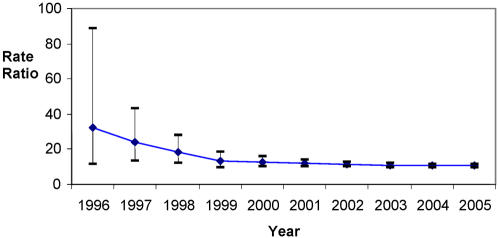
Case-crossover comparison of myopathy/myalgia based on 16,591 users extracted from the THIN database (1991–2006) for all statin and fibrate classes cumulative data of rate ratio (95% CI) by year using 12 week cut off for exposure.

**Figure 3:**
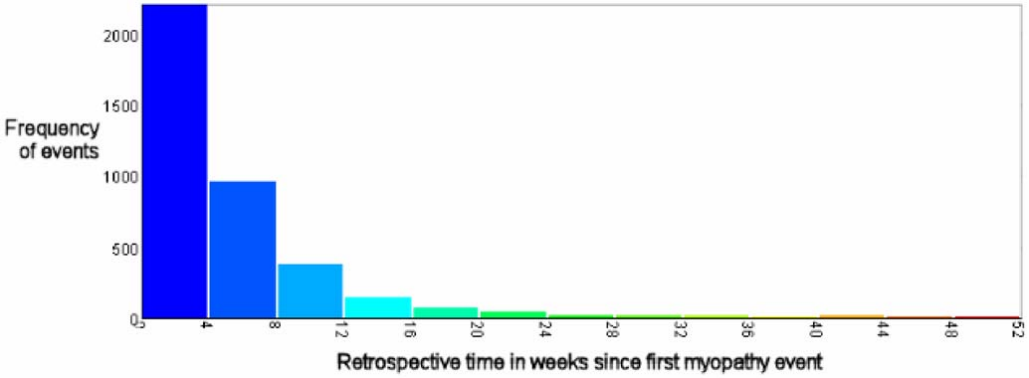
Data from the THIN database 1991–2006: For patients with myopathy following statin: time distribution back to most recent increase or start of new statin.

Figure 2 shows temporal trend of statin associated myopathy from 1996–2006 based on cumulative numbers of exposed and unexposed events (all classes of statin and fibrate). From 1996 it was apparent that the RR for myopathy/myalgia was significantly elevated RR 32.2 (95% CI 11.7–88.6), and the precision increased with time. Figure 3 represents a frequency histogram showing for patients with myopathy following statin: time distribution back to most recent increase or start of new statin. The data shown suggests that most of the cases occur within the first 12 weeks of statin exposure, although a 26 week exposure cut-off will allow less misclassification of exposed and unexposed cases.

## Discussion

This analysis used a case-crossover design to compare risks associated with each class of statin and fibrate. We were able to demonstrate a risk which persisted after exposure and time trend analyses which indicated signals had the potential to be picked up early. Both datasets have independently shown that case-crossover designs are able to identify associations of myopathy related codes. Using 12 week exposures: for IMS data, the rate ratio was 8.2 (95% CI 4.1–16.3) for all myopathy codes and statin exposure. For THIN data, the combined result for all myopathy codes and statin exposure was 10.6 (95% CI 9.8–11.4). Both RRs were of comparable magnitude. Statins are increasingly prescribed which may lead to greater number of events in more recent years, following DOH and NICE recommendations.[24], [25] Furthermore event rates were based on large numbers of exposed compared with unexposed rates which resulted in RR with more precise CI (particularly for classes of drug such as atorvastatin).

The magnitude of the RR with known elevated CK levels for the THIN data for 12 and 26 week cut off for exposure are 8.0 (95% CI 5.8–11.1) and 14.5 (95% CI 7.7–23.7) respectively are lower than for codes for myopathy alone (for all exposure cut-off times) suggesting that not all cases of myopathy had elevated CK levels recorded by their general practitioner, or that not all cases of myopathy have definitively elevated CK levels. Until recently laboratory results had to be manually entered onto the electronic record which is likely to result in the relatively low number of CK values being captured.

Statins have significant health benefits in patients with high risk for cardiovascular disease reducing cardiovascular mortality and morbidity,[3], and serious side effects are rare. When prescribing statins, any drug risk and benefit must be taken into account. For most individuals with high cardiovascular risk, benefits will substantially outweigh any risk from statin prescribing. Graham et al. showed incidence rates of hospitalized rhabdomyolysis to be particularly elevated with combination of statin and fibrate therapy, for example rhabdomyolysis incidence rates for cerivasatin (withdrawn in 2001) was 5.34 (95% 1.46–13.68) and for cerivastatin and gemfibrozil this increased to 1035 (95% 389–2117).[26] We were unable to calculate RR for rhabdomyolysis using the case-crossover design as no events were recorded in the exposed group. Using this UK primary care data, atorvastatin and simvastatin reflect their place as the most commonly prescribed statins and carry similar risk ratios for myopathy for 26 week exposure: 15.2 and 19.5 respectively. Although the largest risks of myopathy were for fluvastatin (RR 33.3 95% CI 16.8–66.0), this is not a widely prescribed statin. The differences between cerivastatin and atorvastatin RR for myopathy were found to be statistically significant at 12 weeks RR 2.05 (95% CI 1.2–3.5) which may reflect a more severe form of myopathy associated with cerivastatin (withdrawn in 2001), than for example atorvastatin or simvastatin. Similarly there were significant differences in RR for myopathy for rosuvastatin and fluvastatin at 12 weeks and 26 weeks RR 3.0 (95% CI 1.6–5.7) and RR 3.4 (95% CI 1.2–8.8), respectively. At 12 weeks, further differences in RR were identified for atorvastatin vs. fluvastatin and pravastatin RR 0.39 (95% CI 0.24–0.63) and 0.53 (95% CI 0.40–0.70); and for simvastatin versus fluvastatin and pravastatin RR 0.44 (95% CI 0.27–0.72) and 0.61 (95% CI 0.46–0.79) respectively; however with longer follow up and larger event numbers these differences may not be significant. Analyses of fibrates prescribed alone showed an overall risk for myalgia/myopathy with all fibrate class (without statin co-prescriptions) was 12.8 (95% CI 6.3–25.9), however this was based on much smaller numbers, and the CI were overlapping for fibrates that had ever been prescribed following statin therapy.

A study in a US managed care group showed the RR of myositis associated with statin monotherapy to be 2.8 (95% CI 1.3–5.9), and combined with fibrate 9.1 (95% CI 3.5–23); however this is unlikely to be representative of the general population.[6] A similar previous study of myopathy using GPRD primary care data showed results of a similar magnitude with ours, comparing patients prescribed statins or fibrates with hyperlipidaemic and normal controls. Although large relative risks for myopathy were found for simvastatin RR 6.1 (95% CI 0.7–56.2) and bezafibrate RR 39.0 (95% CI 9.1–170.0); the precision was lower as the number of cases on which the estimate was based was very small (1–5), and the time period of follow up was shorter: 1991–97.[7]

Using a 26 week exposure cut-off, the number of exposed events 4002 in 350 years in 16, 591 patients suggests an annual incidence of around 11.4 for 16, 591 patients or 689 per million per year, using THIN data, which is much higher than the annual incidence of myopathy[7] or rhabdomyolysis previously estimated with statin monotherapy;[26], [27] however this may be partly due to the fact we have included milder forms of myopathy and myalgia.

Although this study has a number of strengths, there are some possible limitations. THIN and MediPlus provide data on a population of 3 million active (and >5 million historic) patients who are registered with a general practitioner, some with over 20 years of follow up. Comparison of THIN patient-demographic statistics with census data indicates that patients included are representative of the general population. People who are not registered with a GP and temporary residents are not included in this study; however this is a small percentage of the overall study population.

Although statins can now be purchased from a pharmacy without a prescription “over-the counter ” in the UK, this is considerably more expensive than by prescription, accounts for less than 1% of statin sales and is unlikely to have a large effect on the results. To eliminate repeated codes for the same event we analysed re-challenge data as having 6 months without any preceding myopathy code and a statin prescription within the preceding 2 weeks; although it is possible this may be biased if the patient did not report symptoms to the GP in the interim period.

### Confounders and Bias

Other conditions such as rare rheumatic diseases were excluded; however due to the insidious nature of these diseases, these could have been missed if symptoms were sub-clinical. Hence, it is plausible that some cases of myopathy may have been due to undiagnosed rheumatic disease, although we would not expect this to inflate the RR associated with statin induced myopathy greatly. One key advantage of the case-crossover design is that it considerably reduces confounding as each case acts as its own control. Conventional confounders such as age, sex, BMI, and additional existing co morbidities such as renal and liver diseases can therefore be accounted for. However, if these change over time, it may be appropriate to calculate a ‘propensity score’ for the individual which can included in the analyses (for example, diagnoses which may lead to initiation of statin therapy) and to test for confounding by exposure to other drugs. The case-crossover approach is particularly suitable for detecting acute conditions, such as ADRs of relatively acute onset. For case-crossover comparisons the main confounder is any secular trend in prescribing which will give rise to confounding by age.

Analysis examining RR of statin associated myopathy, stratifying by calendar time periods showed possible secular trend of decreasing RR. The adjusted RR (20.5) was similar to the crude estimate of RR; however the fall in RR from 28.7 to 18.8 may be explained by less use of concurrent fibrates with statins. The difference in RR from 1995–98 compared with 2003–5 (12 week data) was found to be statistically significant RR 1.79 (95% CI 1.2–2.8), and was similar for longer periods of exposure (26 weeks) RR 1.53 (95% CI 1.2–2.9).

Where there are large RR associated with drug exposures; any contamination of unexposed with exposed groups will affect the estimates. This misclassification of exposure will mean that many cases classified as “unexposed” are in reality “exposed”. So in effect we expect the true estimate for the RR of statin associated myopathy will be higher than 19.9.

These and other techniques could be used to develop and apply methods for exploiting primary care databases to infer causal relationships between classes of drugs and classes of adverse events. In the longer term, the development of computerised integrated health records could allow the methods to applied to a much wider population and thus greatly improve the detection rates of ADRs. Because of the computerisation of general practice, the UK is well placed to develop these new methods and compare them with existing methods, although other European countries are also adapting to computerised medical records. This would lead to the development and testing of new methods of detecting adverse drug reactions, which if successfully introduced, would have great public health, clinical and economic benefits.

## Supporting Information

Appendix S1 (0.03 MB DOC)
