# Supplementary material for: Statin Induced Myopathy and Myalgia: Time Trend Analysis and Comparison of Risk Associated with Statin Class from 1991–2006
Source: PLoS One. 2008 Jun 25;3(6):e2522. doi: 10.1371/journal.pone.0002522 (PMC2432025; doi:10.1371/journal.pone.0002522)
Supplement: Appendix S1 — (0.03 MB DOC) [file pone.0002522.s001.doc]

***Appendix 1: READ codes used for classification myopathy and related symptoms***

| **READ Code^1^** | **Code Text** |
| --- | --- |
| .F483, .F484, F397., F39z., .F48Z | Myopathy-all codes  Toxic myopathy; proximal myopathy; myopathy unspecified; Myopathy or muscular dystrophy unspecified |
| .M4C1, .M4C2, .M4C3, .M4C6, .M4C7 | Myalgia/myositis –all specified codes |
| N241000 | Myalgia unspecified |
| N241.100 | Myositis unspecified |
| N240z00;N24z.00N241z, .M4CZ, .M4C. | Myalgia/myositis unspecified |
| N23z.00 | Muscle ligament or fascia disorder |
| N241.011  .M4A9, N239., N248. | Intercostal myalgia  Fibromyalgia |
| N240 N240000; N240100;N240200;N240300; | Rheumatism and/or fibrositis unspecified; muscular rheumatism; rheumatic pain |
| N241.012 | Muscle pain |
| 44H4.0 | CK level >1500IU/L M and >1000 IU F |
| SK08. | Acute renal failure due to rhabdomyolysis |

**^1^** Some READ codes have been renamed
